# The nutritional status of Grade 1 pupils, in Bloemfontein, South Africa and its association with socio-demographic data

**DOI:** 10.4102/phcfm.v5i1.475

**Published:** 2013-05-13

**Authors:** Hanneke Brits, Riana Augustyn, Elzette Bezuidenhout, Marisa Cillie, Roelof J.v. Vuuren, Gina Joubert

**Affiliations:** 1 Department of Family Medicine, University of the Free State, South Africa; 2 Department Biostatistics, University of the Free State, South Africa

## Abstract

**Background:**

Despite the fact that UNICEF declared freedom from hunger and malnutrition a basic human right in 1948, more than 20 million children were severely malnourished in 2010 and a further 170 million were stunted. Malnutrition attributes to >50% of child deaths by potentiating infectious diseases.

**Objectives:**

The aim of this study was to determine the extent of malnutrition in Grade 1 pupils in public sector schools in Bloemfontein. An objective of the study was to identify relationships between socio-economic parameters and malnutrition.

**Method:**

Grade 1 pupils from ten public schools in Bloemfontein, selected from a random table, were included in the study. Their parents/caregivers gave informed consent and completed a questionnaire regarding baseline characteristics and feeding practices at home. The children were then weighed and measured, and the 2007 WHO Growth Reference for school-aged children and adolescents used as reference.

**Results:**

A total of 187 children were included in the study. The combination of underweight, wasting and stunting gave an 18% prevalence of malnutrition in this study. A BMI of less than the fifth percentile occurred in 27% of the pupils. Factors positively associated with malnutrition included: Absence of a fridge and/or running water in the house and low education and/or unemployment of parents. Illness in the previous month was reported by 41% of the malnourished children.

**Conclusion:**

As socio-economic factors that contribute to malnutrition are now known, teachers will be able to identify and refer children with or at risk of malnutrition and indirectly decrease child mortality.

## Abstrait

**L’état nutritionnel des élèves de 1re année, à Bloemfontein, Afrique du Sud et son association avec des données socio-démographiques**

**Contexte:**

Bien que l'UNICEF ait déclaré que l’éradication de la faim et de la malnutrition était un droit fondamental en 1948, plus de 20 millions d'enfants étaient sévèrement malnutris en 2010, et 170 millions d'enfants supplémentaires souffraient d'un retard de croissance. La malnutrition est responsable de plus de 50% des décès d'enfants car elle augmente le risque de maladies infectieuses.

**Objectifs:**

L'objectif de cette étude était de déterminer l'ampleur de la malnutrition chez les enfants en première année d’école primaire dans les écoles du secteur public de Bloemfontein. L'un des objectifs de l’étude était d'identifier les relations qui existent entre paramètres socioéconomiques et malnutrition.

**Méthode:**

Les élèves en première année de primaire de dix écoles publiques de Bloemfontein, sélectionnées à partir d'un tableau aléatoire, ont été inclus dans l’étude. Leurs parents/aidants ont donné leur consentement éclairé et ont répondu à un questionnaire traitant des caractéristiques de référence et les pratiques alimentaires à la maison. Les enfants étaient ensuite pesés et mesurés, et la Référence de croissance pour les enfants d’âge scolaire et les adolescents de l'OMS a été utilisé comme référence.

**Résultats:**

187 enfants ont été inclus dans l’étude. La combinaison d'insuffisance pondérale, d'amaigrissement et de retard de croissance indiquait une prévalence de malnutrition de 18% dans cette étude. Un IMC inférieur au cinquième percentile était observé chez 27% des élèves. Les facteurs positivement associés à la malnutrition incluaient: l'absence de réfrigérateur et/ou d'eau courante à la maison et le faible niveau d’éducation et/ou le chômage des parents. Une maladie au cours du mois précédent a été rapportée par 41% des enfants malnutris.

**Conclusion:**

Maintenant que les facteurs socioéconomiques contribuant à la malnutrition sont connus, les enseignants pourront identifier et orienter les enfants souffrant de malnutrition ou exposés à un risque de malnutrition et réduire indirectement la mortalité infantile.

## Introduction

In the 1948 Universal Declaration of Human Rights the United Nations International Children's Emergency Fund (UNICEF) declared freedom of hunger and malnutrition a basic human right.^1^ Despite this, 104 million children worldwide under the age of five years were underweight for age in 2010; 171 million were stunted and a further 20 million suffered from severe malnutrition.^2^

### Background

The World Health Organization (WHO) defines malnutrition as ‘the cellular imbalance between supply of nutrients and energy and the body's demand for them to ensure growth, maintenance, and specific functions’.^3^ It is important to realise that malnutrition is not only a term used to indicate poor nutritional status, but that according to a study by Blossner and de Onis4 malnutrition also attributed to 54% of child deaths by potentiating infectious diseases. Most of these deaths (83%) occurred in mildly and moderately malnourished children and not in the severely malnourished group.

A recent study by Armstrong et al^5^ as well as a study in Sri Lanka^6^ demonstrated a relationship between socio-economic status and malnutrition in pre-school children in developing countries. Several studies^6, 7, 8^ linked malnutrition with low income, unemployment, low educational status of parents, poor sanitation, type of housing and number of people per household.

If teachers are aware of the specific risk factors for malnutrition they can identify these children and also focus their education programmes on practical issues regarding diet and nutrition. In this way they can indirectly assist in reducing child mortality.

Malnutrition in children attributes to 54% of child deaths by potentiating infectious diseases. This study identified risk factors for malnutrition in Grade 1 pupils and will therefore enable teachers to refer and support children at risk and thereby indirectly contribute to a decrease in child mortality.

#### Aim and objective

The primary aim of this study was to determine the extent of malnutrition and particularly under-nutrition in Grade 1 pupils in public sector schools in Bloemfontein. An objective of the study was to identify relationships between socio-economic parameters and malnutrition.

#### Significance of the study

Malnutrition is a preventable and treatable condition, but it can have serious consequences if it is not managed adequately. If the risk factors for malnutrition are known, teachers will be able to identify children at risk and refer them for proper treatment. Schools will also be able to implement feeding programmes for the children with malnutrition as well as those at risk.

## Research methods and design

### Population

Grade 1 pupils from six to seven years of age, attending public schools in Bloemfontein were our study population. From the 46 public schools in Bloemfontein (5893 Grade 1 pupils), 10 were selected using a random number table. One Grade 1 class per school was then chosen for the study according to a random number table. The exclusion criterion was Grade 1 pupils who had not obtained consent from the school and/or a parent/guardian.

### Setting

Bloemfontein is situated in the centre of South Africa and is the capital city of the Free State Province. Together with Botshabelo and Thaba Nchu it forms the metro of Mangaung.

### Design

This was a cross-sectional study where Grade 1 pupils were weighed and measured to obtain their anthropometric data and parents or caregivers supplied demographic information by means of filling in a questionnaire.

### Data collection methods and tools

A pilot study was conducted at one school to test the questionnaires. Twenty-five questionnaires were filled in and returned. The questionnaire was then changed slightly and therefore the initial results were not included in the actual study. Validity was accepted at face value.

Thereafter the principals of each of the selected schools were contacted and the study was explained to them. All gave their written consent for the study. The consent forms and questionnaires developed by the researchers were distributed to the selected Grade 1 pupils. Consent forms as well as questionnaires were supplied in the learner's language of choice – Afrikaans, English or Sotho – to be completed by parents or guardians at home. The questionnaires were returned to the school in sealed envelopes to protect confidentially. In this way demographic data, as well as information on the number of people and children per household, type of housing, sanitation and availability of water, electricity and fridges were collected. Information regarding the child's food consumption and the parents’ education level was also obtained. On the pre-arranged day the researchers then weighed and measured all the children who had obtained consent for the procedure and noted their measurements on the questionnaires. Standardised methods were used for the weight and height of each child, with a calibrated scale and a non-stretchable measuring tape attached to a wall. Data were collected during 2006 and analysed only after the 2007 guideline became available.

## Ethical considerations

The study was approved by the Ethics Committee of the Faculty of Health Sciences of the University of the Free State as well as the Department of Education in the Free State (ETOVS 23/04).

In 2006 WHO published the WHO Child Growth Standards,^9^ which is regarded as the standard to measure the nutritional status of preschool children. The WHO Growth Reference for school-aged children and adolescents^10^ was published in 2007 and it was used as reference in this study, together with body mass index (BMI) charts specific for age and gender. The following definitions, quoted from the above guidelines, were used in this study:

- **Underweight:** Weight-for-age less than two standard deviations below the median reference value. Underweight reflects a combination of acute and chronic malnutrition.
- **Wasting:** Weight-for-height less than two standard deviations below the median reference value. Wasting is considered an indication of acute or recent malnutrition.
- **Stunting:** Height-for-age less than two standard deviations below the median reference value. Stunting is considered an indication of chronic malnutrition.
- **Overweight:** A BMI above the 85th percentile and lower than the 95th percentile for children of the same age and sex.
- **Obesity:** A BMI at or above the 95th percentile for children of the same age and sex****.

The body mass index (BMI) for age and sex is calculated as weight (kg)/height (m^2^). A BMI of less than the fifth percentile is considered as underweight according to WHO as well as the Centers for Disease Control and Prevention (CDC).

Analysis of the data was carried out by the Department of Biostatistics, UFS. Weight-for-age, height-for-age and weight-for-height tables and charts were used to differentiate between acute and chronic malnutrition. Z-scores of less than minus two SDs were regarded as malnutrition. Subgroups were compared, using chi-squared or Fisher's exact tests for categorical data and median tests for skew numeric data.

## Results

A total of 187 children were included in the study sample giving a response rate of 67%. The sample consisted of 55% (103) males and 45% (84) females. No child in this study was overweight-for-age or obese.

The results of the categories of malnutrition, namely weight-for-age, height-for-age, weight-for-height and gender are displayed in Table 1. Weight-for-age is used to make the diagnosis of underweight, weight-for-height for wasting and height-for-age for stunting. No statistically significant difference occurred between boys and girls, with all *p*-values >0.05. Nine per cent of the pupils were underweight.

**TABLE 1:** Relationship between gender and weight categories.

Categories	Boys		Girls		*p*-value
			
	*n*	%	*n*	%	
Underweight	11	11	6	7	0.43
Wasting	13	13	8	10	0.54
Stunting	4	4	3	4	0.93

Stunting, which indicated chronic malnutrition, occurred in four per cent of the children. A weight-for-height calculation of minus two to minus three of the SD for median reference value, indicative of acute weight loss, occurred in 11% of the children. A total of 34 pupils (18%) presented with at least one of the three forms of malnutrition. A BMI of less than the fifth percentile occurred in 27% of the pupils.

The presence of running water to prepare hygienic food and a fridge to store the food safely was associated with less malnutrition as displayed in Table 2. A *p*-value of <0.05 was indicative of a statistically significant difference between two measurements. This information was not available for all the children; it was only available for 153 fathers and 168 mothers. The information was, however, available for all the malnourished children.

**TABLE 2:** The association of malnutrition with the presence of running water, a flush toilet and a fridge in the house.

House	Variables	Children (*n*)		*p*-value
			
		Without malnutrition	With malnutrition.	
Running water	Water in house	98	11	0.01
	No water in house	55	23	
Flush toilet	Flush toilet	134	28	0.17
	No flush toilet	19	6	
Fridge	Fridge in house	137	24	0.04
	No fridge in house	18	10	

The majority of the children ate breakfast (82%), lunch (92%) and dinner (96%) on a daily basis and these factors were not associated with malnutrition. Twenty-four children did not take food to school and did not eat snacks between meals; 50% of these children were malnourished. This factor had a significant association with malnutrition with a *p*-value of 0.007.

The positive association between malnutrition and unemployment and poor education is demonstrated in Table 3.

**TABLE 3:** Association of malnutrition in Grade 1 pupils with parents’ employment and educational status.

Association	Status	Children without malnutrition		Children with malnutrition		*p*-value
				
		*n*	%	*n*	%	
Father	Employed	91	76	17	50	0.047
	Unemployed	28	24	17	50	
Mother	Employed	78	58	12	35	0.0002
	Unemployed	56	42	22	65	
Father	With Grade 12	73	61	13	40	0.0177
	< Grade 12	46	39	21	60	
Mother	With Grade 12	79	59	11	32	0.0024
	< Grade 12	55	41	23	68	

Illness during the previous month was reported by 41% of the malnourished children. This factor was significantly associated with stunting (*p* = 0.02), wasting (*p* = 0.04) and being underweight (*p* = 0.02).

The ownership of a house, type of house, number of people in the house, number of children in the household and sanitation method could not be associated with malnutrition in this study.

## Discussion

The term malnutrition includes both overweight and underweight. As no child included in this study was overweight, the discussion will only focus on under nutrition. Different classifications and calculations are used to look at different types of malnutrition and they will also be discussed in this study.

The 9% prevalence of underweight (measured as weight-for-age) in Grade 1 pupils in Bloemfontein is comparable to the 14% for the Free State which was recorded by the National Food Consumption Survey (NFCS-FB_I) of 2005, if the fact that this study was performed in an urban area is taken in consideration,^11^ but is less than the 30% prevalence of underweight in sub-Sahara Africa and the global prevalence^12^ of 17.6%. In the United States of America the incidence of underweight is less than 1% and in India it is more than 50%.^4^

The association of malnutrition with the absence of water and fridges was statistically significant in this study population. The presence of water in the house provides better opportunities for the hygienic preparation and consumption of food and a fridge keeps food fresh for longer periods. This may also be an indirect measurement of improved socio-economic circumstances.

It was encouraging to see that most children ate at least three times a day. A concern is the 34 children (18%) who did not eat breakfast before they went to school, although this factor did not contribute to malnutrition. However, the recommendation for children of this age is three meals plus two to three snacks a day.^13, 14^ The 50% prevalence of malnutrition in children who did not take food to school or eat snacks between meals is something that needs to be addressed as nutritional snacks may improve the children's nutritional status. The type and amount of food that the children ate and took to school were not identified in this study.

Parents’ low education and unemployment levels were significantly associated with malnutrition. It needs to be kept in mind that education and employment provide opportunities to earn money and to support the family. Unemployment and/or low education levels in mothers were identified in the study as important factors contributing to malnutrition. Mothers’ low education levels as a cause of malnutrition was demonstrated in numerous studies.^6, 15, 16, 17^ Employment and education of parents were identified as protective factors against malnutrition in this study. ‘Educate the girl child to save the next generation from malnutrition’.^6^

Illness during the previous month was identified as a major contributing factor in malnutrition. Illness during the previous month occurred in 41% of the malnourished children. It is, however, difficult to determine causes and results here, as malnutrition can lead to suppressed immunity, which can lead to disease and as disease can cause malnutrition because of poor food intake during illness. Although BMI is used more often to diagnose and monitor overweight, it can also indicate rapid weight loss as weight changes occur more rapidly than changes in length over a short period of time, e.g. during a time of acute illness. A BMI of less than the fifth percentile, which occurred in 27% of the pupils, may be an indication of acute weight loss because of an illness during the previous month, rather than of chronic malnutrition, as the latter occurred only in five per cent of the children.

The ownership of a house, type of house, number of people in the house, number of children in the household and sanitation method could not be linked to malnutrition in this study. It has, however, been linked to malnutrition in studies performed in Sri Lanka^6^ and Bangladesh.^7^ A study in China^18^ did not find a relationship between the number of children in the household and malnutrition, either.

## Limitations of the study

The response rate of 67% gave us a confidence interval of 7.1% and a standard error of 3.6% which is a little low, but still acceptable for this study at a confidence level of 95%.

Standardised methods and equipment were used to ensure accurate measurements, but measurement errors were still possible.

The questionnaires did not include information on the type or amount of food that was given for meals or snacks, but only on how often food was eaten.

Although the questionnaires were anonymous the accuracy of the information supplied could not be guaranteed.

It was difficult to compare the results of different studies as different parameters for malnutrition were used in different studies and some studies did not indicate which measurement/s they used.

### Recommendations

1. Grade 1 teachers should know the risk factors for malnutrition and identify and appropriately refer these children.
2. Feeding programmes at schools should focus on children at risk and also on snacks between meals.
3. The Department of Education should adjust the school curriculum so that the subject of a healthy diet is introduced from the beginning of the school years. The Department should also keep children in school to improve their education and to prevent malnutrition in future.
4. Unemployment and the lack of water and electricity in households should be priorities for local, provincial and national government.

It bears repeating: ‘Educate the girl child to save the next generation from malnutrition’.^6^

## Conclusion

Although the 18% prevalence of malnutrition in our study population was within the global norms, it means that one in every five Grade 1 pupils suffers from malnutrition. This makes them more prone to infections, which, in turn, can lead to early morbidity.

Different factors influence malnutrition in different parts of the world. In the Bloemfontein region the absence of water and fridges in houses, parents’ unemployment and low education levels and the fact that children did not take food to school were the major factors associated with malnutrition. Illness during the previous month was also strongly associated with malnutrition.

## References

[1] A UNICEF Policy Document Strategy for improved nutrition of children and woman in developing countries. October 1992 World Health Organization: Malnutrition - The Global Picture [homepage on the Internet]. 2000 [Cited 2009 Mar 15]. Available from: http://www.who.int/home-page

[2] WHO Fact sheet on child morbidity and mortality. [homepage on the Internet]. 2010 [Cited 2012 June 21]. Available from: http://www.who.int/mediacentre/factsheets/fs178/en/

[3] World Health Organization; UNICEF; UN System Standing Committee on Nutrition (2006). WHO, UNICEF, and SCN informal consultation on community-based management of severe malnutrition in children – SCN Nutrition Policy Paper No. 21 [homepage on the Internet]. No date [Cited 2009 Mar 15]. Available from: http://www.who.int/child_adolescent_health/documents/fnb_v27n3_suppl/en/index.html

[4] Blossner M, De Onis M Malnutrition: quantifying the health impact at national and local levels. Environmental Burden of Disease Series. Geneva, Switzerland: WHO; 2005.

[5] Armstrong J, Dorosty AR, Reilly JJ Co-existence of social inequalities in undernutrition and obesity in preschool children: Population based cross sectional study. Arch Dis Child 2003;88:671–675. http://dx.doi.org/10.1136/adc.88.8.671, PMid:12876159, PMCid:1719615 12876159 10.1136/adc.88.8.671 PMC1719615

[6] Nutritional status of pre-school children in Sri-Lanka. Department of Census and Statistics. Asian Development Bank Presented at the ADB/UNESCAP concluding Workshop on Enhancing Social and Gender Statistics, Bangkok, 2003 June 24–27.

[7] Pryer JA, Rogers S, Rahman A The epidemiology of good nutritional status among children from a population with a high prevalence of malnutrition. Public Health Nutr. 2003;7(2):311–317. 15003139 10.1079/PHN2003530

[8] Nandy S, Irving M, Gordon D, Subramanian SV, Smith GD Poverty, child undernutrition and morbidity: New evidence from India. B World Health Organ. 2006;83(3):210–216. PMC2624218 15798845

[9] WHO Child growth standards [homepage on the Internet]. 2006 [Cited 2009 March 12]. Available from: http://www.who.int/childgrowth/standards/en/

[10] WHO Growth reference data for 5-19 years [homepage on the Internet]. 2007 [Cited 2009 March 12]. Available from: http://www.who.int/growthref/en/

[11] Combating malnutrition in South Africa [homepage on the Internet] 2008 [Cited 2009 March 12]. Available from: http://www.dbsa.org/Research/Documents/South%20Africa%20Nutrition_%20input%20paper_roadmap.pdf

[12] WHO Malnutrition - The Global Picture. World Health Organization [homepage on the Internet]. No date [Cited 2009 March 12]. Available from http://www.who.int/home-page/

[13] Brown C Integrated nutrition programme for the Department of Health in South Africa [homepage on the Internet]. No date [Cited 2012 June 21]. Available from: http://www.doh.gov.za/docs/programmes/2010/guide.pdf

[14] Handbook of IMCI Integrated Management of Childhood Illness Counsel the mother on feeding and fluids. WHO publication, 2005; p 119–126.

[15] Christiansen L, Alderman H Child malnutrition in Ethiopia: Can maternal knowledge augment the role of income? October 2001. African Region Working Paper Series No. 22.

[16] Oyekale AS, Oyakale TO Do mothers’ educational levels matter in child malnutrition and health outcomes in Gambia and Niger? [homepage on the Internet]. No date [Cited 2009 March 15]. Available from: http://www.saga.cornell.edu/saga/educconf/oyekale.pdf

[17] Griffiths P, Madise N, Whitworth A, Matthews Z A tale of two continents: a multilevel comparison of the determinants of child nutritional status from selected African and Indian regions. Health Place 2004;10:183–199. http://dx.doi.org/10.1016/j.healthplace.2003.07.001, PMid:15019912 15019912 10.1016/j.healthplace.2003.07.001

[18] Hesketh T, Qu JD, Tomkins A Health effects of family size: Cross sectional survey in Chinese adolescents. Arch Dis Child 2003;88:467–471. http://dx.doi.org/10.1136/adc.88.6.467, PMid:12765907, PMCid:1763126 12765907 10.1136/adc.88.6.467 PMC1763126

